# Evaluation of a Remote Symptom Assessment and Management (SAM) System for People Receiving Adjuvant Chemotherapy for Breast or Colorectal Cancer: Mixed Methods Study

**DOI:** 10.2196/22825

**Published:** 2020-12-07

**Authors:** Lisa Whitehead, Laura Emery, Deborah Kirk, Diane Twigg, Deborah Brown, Joanna Dewar

**Affiliations:** 1 Edith Cowan University Joondalup Australia; 2 Sir Charles Gairdner Hospital Perth Australia

**Keywords:** self-management, intervention, symptom management, breast cancer, colorectal cancer, cancer, symptom, monitoring, online intervention, development, implementation, evaluation

## Abstract

**Background:**

The Symptom Assessment and Management (SAM) program is a structured, online, nurse-supported intervention to support symptom self-management in people receiving adjuvant chemotherapy post surgery for breast or colorectal cancer.

**Objective:**

The objective of this study was to describe the development, implementation strategy, and evaluation of the SAM system.

**Methods:**

The development of the SAM program involved 3 phases. In phase 1, the web app was developed through consultation with consumers and clinicians and of the literature to ensure that the system was evidence-based and reflected the realities of receiving treatment and supporting patients through treatment. In phase 2, 7 participants recorded the severity of 6 symptoms daily over the course of 1 cycle of chemotherapy. In phase 3, 17 participants recorded their symptoms daily over the course of 3 cycles of chemotherapy. Once symptoms were recorded, participants received immediate feedback on the severity of their symptoms and self-management recommendations, which could include seeking immediate medical attention. Data on quality of life, symptom burden, anxiety and depression, distress, and self-efficacy were collected during treatment; participants’ perceptions of the SAM program were evaluated following participation via interview.

**Results:**

The outcomes of the SAM project include the development of a system that is reliable and easy to use and navigate. Participants reported benefits related to using the SAM program that included feeling more in control of managing their symptoms and feeling reassured. Engagement with the system on a daily basis was variable, with some participants completing the symptom tracker daily and others engaging some of the time. The feedback from all participants was that the system was easy to navigate and the information was relevant and supportive.

**Conclusions:**

The SAM program has the potential to enhance the management of symptoms for people receiving chemotherapy treatment. The system creates an accurate repository of symptoms that can be accessed easily and highlight patterns in symptom experience. These can be shared with clinicians, with patient permission, to inform and support treatment plans. The potential to predict the risk of developing severe symptoms can be developed to anticipate the need for care and support. Further considerations on how to increase engagement with the system, the value of the system for people diagnosed with other tumor types and treatment regimes, and the incorporation of the system into everyday clinical practice are needed.

## Introduction

Chemotherapy is a core component of cancer care for many people diagnosed with cancer and can be used as an adjuvant treatment, which means chemotherapy is an additional cancer treatment given after the primary treatment, usually surgery, to lower the risk that the cancer will return. There are a range of symptoms commonly experienced by people receiving chemotherapy, including pain, fatigue, trouble sleeping, nausea, vomiting, distress, anxiety, and depression [1]. The experience of symptoms can have an impact on the ability to adhere to treatment regimens and on quality of life [2,3]. In addition, the toxic effects of chemotherapy can be serious and life-threatening; for example, dehydration following vomiting and/or diarrhea and infection following leukopenia. Chemotherapy is most often delivered in the outpatient setting and the majority of associated side effects are managed in the community setting. The ability to communicate with health care providers in a timely way about symptoms that are impacting daily functioning, have become moderate or severe, or are prolonged is important to promote effective self-management and prevent hospitalization, or, in the case of severe or prolonged symptoms, advise on the need for urgent attention [1,2,4].

Technology can facilitate both the monitoring of symptoms and communication between patients and health care providers, and there has been a rapid increase in the number of systems in development; however, many require further development in order to enhance their usability and clinical integration [5], and few studies have been conducted in cancer settings [6].

An increasing number of mobile apps have been designed to support cancer care. Charbonneau et al [7] found 123 digital health options specific to cancer patients. These apps provide a variety of services for those in the cancer community. The apps support self-reporting and home monitoring of symptoms associated with cancer treatment [2,8-14] and report toxicities directly to the provider [2,8-12,14], as well as provide disease-specific monitoring [10,11], monitoring during active phases of cancer care [2,8,9,14] or during hospice stays [12], evidence-based education or self-care advice based on patient input to the system [2,12], and/or community cancer resources [2,15].

The web app developed and implemented in this study, the Symptom Assessment and Management (SAM) system, combined all of these features with the addition of an alert system. Based on patient scores, or levels of toxicity, alerts were generated ranging from evidence-based education to contact with providers to emergency assistance. We believe that the web app, developed in collaboration with consumers and health care providers, provides a comprehensive platform for people receiving adjuvant chemotherapy for breast or colorectal cancer to access information, assess and chart a range of symptoms, and receive real-time self-care advice.

## Methods

### Study Aim

The aim of the pilot study was to develop and implement a SAM web app for patients receiving chemotherapy as adjuvant treatment after surgery for breast or colorectal cancer. Participants did not have a diagnosis of active cancer. Participants were prompted by the mobile, web-based system to assess symptoms during treatment and they were provided with evidence-based real-time recommendations to support their self-management.

### Study Design

A mixed methods design was employed involving 3 phases: (1) phase 1, development of the web app, (2) phase 2, pilot involving one cycle of chemotherapy, and (3) phase 3, intervention over 3 cycles of chemotherapy.

#### Phase 1: Development Phase

In the development phase, a review of the literature related to best practice of symptom management was conducted to develop alert levels for symptom severity and self-care messages. The guidelines and self-management documents highlighted the most commonly reported symptoms, when patients need to contact a clinician for advice or seek emergency support and self-management advice on managing symptoms. Following the review, consultation with consumers who had experience of breast or colorectal cancer and meetings with clinicians were held to further inform the selection of symptoms to be included in the system, the development of the alert algorithms and the content of the self-care messages. The symptoms of nausea, vomiting, diarrhea, constipation, temperature, mouth and throat sores (mucositis), neuropathy (numbness in hands and feet), fatigue, and distress were selected. An external company was employed to design and create the interactive web platform, the SAM platform. Three meetings were held with the developers to discuss the content to be included as supplied by the research team, usability features of the web app, presentation of the web app and pretesting. In addition to tracking symptoms on a daily basis, the web app provided participants with relevant phone numbers, self-care advice, access to evidence-based resources, and a summary page of symptoms (my symptom history). Participants were also reminded that the site was “not monitored 24/7 and should not be used as a replacement for medical appointments.” An advisory committee was established and included clinicians from the medical oncology team, nursing representatives, the research team, and three consumer representatives who had experienced cancer and received chemotherapy. Two meetings were held to report on progress and seek advice on recruitment strategies.

#### Phase 2: Pilot

In Phase 2, 7 participants were asked to record their symptoms daily on the web app for 1 chemotherapy cycle using either a personal computer, iPad (Apple Inc), or smartphone. Participants were invited to record their temperature using a thermometer provided by the research team to check whether they had a raised temperature, which could be indicative of an infection. Participants completed questionnaires at baseline (prior to the chemotherapy cycle) and again at the commencement of cycle 2 (approximately day 14 for colorectal cancer patients or approximately day 21 for breast cancer patients) to evaluate quality of life, symptom burden, mental health, and self-efficacy. Participants completed an interview at the end of the cycle of chemotherapy. The key aim of this phase was to ensure that the web app was functional from a user perspective, data were collected and stored as planned, and the alert system did not create undue distress for participants or additional and unnecessary demands on health care staff.

#### Phase 3: Intervention

In phase 3, 17 participants recorded symptoms on the web platform over 3 chemotherapy cycles. Participants were also invited to record their temperature using the thermometer provided to check whether they had a raised temperature, which could be indicative of an infection. During this phase, participants completed questionnaires at baseline (prior to the chemotherapy cycle) and at the commencement of cycles 2, 3, and 4. Interviews were conducted at the end of cycle 3. The aim of this phase was to explore the usability and utility of the site over a longer period of time, including the number of alerts generated and the actions taken as a result of the alerts generated.

### Ethics

The study received approval from the human research ethics committee at the hospital as well as the university ethics committee.

### The SAM System

The web-based system comprised four functions: (1) to monitor symptoms experienced on a daily basis or anytime a participant wanted to assess their symptoms, (2) to provide immediate feedback on self-care actions to be taken based on the data entered, (3) to map symptoms on a graph over time that could be reviewed by the participant or provided to clinicians to review, and (4) to provide a repository of evidenced based information on key symptoms for further reading and consultation.

The decision to build a web-based app over a native app was driven primarily by the goals of the system and secondly by cost. The web-based app was able to meet all of the study needs and was able to be viewed and used across a range of devices (desktops computers, laptops, iPads, and smartphones). The research team believed that a native app would not add any benefit in relation to aesthetics or functionality but would risk compromising accessibility and would cost substantially more to create. A screenshot of the log-in page as it appears on a smartphone and a tablet are presented in Multimedia Appendices 1 and 2, and additional screenshots, including examples of self-care messages, are presented in Multimedia Appendices 3-5.

#### Daily Symptom Monitoring

The system allowed for real-time symptom monitoring and management of nausea, vomiting, diarrhea, constipation, mouth and throat sores (mucositis), neuropathy (numbness in hands and feet), fatigue, and distress in patients who had surgery for early breast or colorectal cancer. A scale from 0 to 10 was created for each symptom, allowing participants to slide a cursor up and down the scale. On completion of the scales, a series of self-care messages were sent back to guide self-care, and within these messages was the alert level that the scores had generated. The algorithms differed by symptom, but self-care advice was either green (no or mild symptoms), amber (indicating an area of concern), or red (indicating serious concern and the need to take action). For example, an amber alert would advise participants to contact their medical oncology team or general practitioner during business hours or, if the participant was concerned, to visit their closest emergency department after hours; a red alert would suggest that the participant go to their closest emergency department for immediate assistance. As per the study protocol, red alerts were forwarded to the medical oncology clinical nurse manager (CNM) within 1 to 3 days of being received by the research team, in case additional follow-up was required. The symptoms were mapped onto graphs accessible on a separate page on the site and these allowed participants to view the trajectory of symptoms over time.

#### Library of Resources

A library of resources was generated to include links to one or more evidence-based sites for each symptom. The resource page was referred to in the self-care messages where appropriate and was accessible at all times to participants to access when necessary. The contact numbers of key personnel and groups were displayed in two places on the site: on the home page and on the self-management report page following the entry of symptoms.

### Population and Setting

Participants were individuals who had received a diagnosis of breast or colorectal cancer, completed surgery, and were scheduled to receive adjuvant chemotherapy at a tertiary hospital in a metropolitan area. Additional inclusion criteria were that participants were aged 18 years or older; receiving a minimum of 3 cycles of chemotherapy on an outpatient basis; able to read, write, and speak English sufficiently well to participate in data collection; deemed by a member of the health care team to be physically and psychologically fit to participate in data collection; able to provide written consent (hard copy or electronic); and willing and able to use their own computer, iPad, or smartphone with internet access to complete the study.

The exclusion criterion was a prior experience of chemotherapy. 

### Participant Recruitment

Participating medical oncologists and the CNM identified eligible participants by screening patient referrals and medical history prior to chemotherapy clinics each week. Those eligible to participate were given information about the study by the participating medical oncologists or CNM and offered a copy of the participant information and consent form to review. For patients who agreed, their details (name, phone number, date of first chemotherapy cycle) were forwarded to the research assistant and contact was made following a chemotherapy education session. Consenting participants were shown face to face how to access and navigate the site and/or emailed information to help them access the SAM website and a link to complete the surveys at each time point. Prior to the start of phase 1, medical oncology and nursing staff were invited to attend a short training session on the recruitment process and how the SAM web portal would be used by participants. The goal was to recruit up to 10 people in phase 2 to test the system across one cycle of chemotherapy and to recruit up to 40 people in phase 3. Because this was a feasibility study without a comparison group, the figures were based on how many participants it seemed reasonable to recruit within the study time frame and based on a review of the outpatient list of potentially eligible patients over the preceding month.

### Participant Questionnaires

Participants completed the Functional Assessment of Cancer Therapy-Breast (FACT-B) [16] and Functional Assessment of Cancer Therapy-Colorectal (FACT-C) [17] cancer scales to measure quality of life, the Rotterdam Symptom Checklist (RSCL) to measure symptom burden [18], the Hospital Anxiety and Depression Scale (HADS) to assess mental health [19,20], and the Strategies Used by Patients to Promote Health (SUPPH) [21,22] to measure self-efficacy. The FACT-B has been assessed as appropriate for use in oncology clinical trials, as well as in clinical practice. Ease of administration, brevity, reliability, validity, and sensitivity to change have been reported [16]. Significant sensitivity to change in the performance status rating was demonstrated for the FACT-B total score, the physical well-being subscale, the functional well-being subscale, and the breast cancer subscale. An alpha coefficient (internal consistency) for the FACT-B total score has been reported to be high (α=.90), with subscale alpha coefficients ranging from .63 to .86. There is evidence to support test-retest reliability, as well as convergent, divergent, and known groups validity. The reliability and validity of the FACT-C was reported across three samples that differed based on the extent of disease and ethnicity [17]. Across the samples, adequate reliability and validity were demonstrated for the FACT-C. Internal consistency analyses across the samples yielded alpha coefficients above .85 for the FACT-C total score. The FACT-C was able to distinguish among patients of different functional categories, particularly between ambulatory patients and patients who required bed rest for some period of time during the day. For patients whose functional status worsened, their quality of life worsened compared with patients whose functional status stayed the same or got better, indicating that the FACT-C is sensitive to changes in functional status.

For the RSCL, the reliability of the three subscales is high, with alpha coefficients ranging from .80 to .87 on the physical symptom distress scale, .85 to .94 on the psychological symptom distress scale, and .86 to .95 on the activity level scale [23]. The clinical validity of the RSCL is reported as satisfactory. The physical distress scales, subscales, and individual physical items differentiate between disease and treatment states as well as moments of treatment process. The psychological scale differentiates between cases and noncases [23].

The HADS has demonstrated satisfactory psychometric properties across a range of groups: in primary care [20], with cognitively intact patients in nursing homes [24], with inpatients with cancer [25], and in the general population [20,26].

The SUPPH was developed to measure patients’ confidence in carrying out self-care strategies [21]. Good initial psychometric properties were found in patients receiving either cancer chemotherapy or hemodialysis. The alpha coefficients were well above the desired criterion of .70. Test-retest and generalizability estimates for the SUPPH were high [22].

Measures were completed at baseline and at the commencement of cycle 2 (phases 2 and 3) and cycles 3 and 4 (phase 3 only). Participants were emailed a survey link at each time point, which allowed them to complete the questionnaires through the Qualtrics online survey platform [27]. This ensured that participants received their questionnaires independent of clinic visits and it allowed questionnaires and reminders to be emailed directly to participants by the research team without the need to involve clinical staff. If requested, paper questionnaires were mailed to participants who preferred to complete the questionnaires in hard copy. 

### Interviews

Participants were invited to participate in a semistructured phone interview to explore their experiences using the web platform. The question guide used in the interviews was developed by the research team in consultation with the consumer advocates and advisory committee. Interviews were transcribed and analyzed using content and thematic analysis to identify common themes. Questions addressed the relevance of items in the scale (symptoms), the self-care messages, the resources section, and the symptom graphs; the experience of any symptoms not included in the scale; and feedback on the layout of the site and navigation and recommendations to improve the site. 

### Data Analysis

Data from the demographic questionnaire and questionnaires retrieved from Qualtrics were saved for analysis using SPSS software (IBM Corp). Data analysis explored changes over time in relation to quality of life, symptom burden, mental health, and self-efficacy. Phone interviews were transcribed using NVivo software (QSR International) and analyzed using content and thematic analysis. Content analysis of the comments created within the system were undertaken where they were generally short and supported the reason for recording a certain symptom severity. Thematic analysis [28] was undertaken with the interview data involving the stages of familiarization, coding, generating initial themes, reviewing themes, designing and naming themes, and writing up.

## Results

### Sample Characteristics

In total, 44 individuals were approached to participate in the study and 24 of them consented to participate. The sample comprised 12 women who had been diagnosed with breast cancer (phase 2, n=3; phase 3, n=9) and 12 people diagnosed with colorectal cancer (phase 2, n=4; phase 3, n=8). No participant had active disease or was scheduled to receive adjuvant chemotherapy. Table 1 sets out the demographic details.

**Table 1:** Sociodemographic characteristics of study participants.

Characteristics			Phase 2 (n=7)			Phase 3 (n=17)		Total (N=24)	
**Age (years)**									
	≤29, n (%)			0 (0)			1 (6)		1 (4)
	30-49, n (%)			2 (29)			4 (24)		6 (25)
	≥50, n (%)			5 (71)			12 (71)		17 (71)
	Mean (SD)			57.4 (11.7)			55.2 (14.8)		55.8 (13.8)
**Cancer diagnosis, n (%)**									
	Breast			3 (43)			9 (53)		12 (50)
	Colorectal			4 (57)			8 (47)		12 (50)
**Gender, n (%)**									
	Male			0 (0)			5 (29)		5 (100)
	Female			7 (100)			12 (71)		19 (100)
**Marital status^a^, n (%)**									
	Married/partnered			4 (57)			10 (59)		14 (58)
	Not partnered (single/separated/divorced)			2 (29)			7 (41)		9 (38)
**Country of birth, n (%)**									
	Australasia			5 (71)			10 (59)		15 (63)
	Europe			1 (14)			5 (29)		6 (25)
	South Africa			1 (14)			0 (0)		1 (4)
	Asia			0 (0)			2 (12)		2 (8)
**Highest level of education, n (%)**									
	Completed high school			4 (57)			5 (29)		9 (38)
	Trade or technical and further education certificate			0 (0)			4 (24)		4 (17)
	Tertiary qualification/s			3 (20)			8 (47)		11 (46)
**Employment status^b^, n (%)**									
	Full-time			1 (14)			5 (29)		6 (25)
	Part-time/casual			2 (29)			6 (35)		8 (33)
	Homemaker			1 (14)			1 (6)		2 (8)
	Retired/not working			3 (43)			5 (29)		8 (33)

^a^Values do not always total 100% due to missing responses for some variables.

^b^Within 12 months prior to diagnosis.

### Engagement with the Web Platform (SAM)

#### Visits to the Website and Completion of the Symptom Tracker: Phase 2

The web analytics allowed tracking of the number of visits to the website, pages visited, completion of the symptom tracker, and dates for each. The number of times the symptom tracker was completed in phase 2 ranged from 9 to 33 (days enrolled in SAM ranged from 15 to 67 days). The number of missed days ranged from 0 to 34 days, a completion rate that ranged from 51% to 100% of days symptoms were recorded using the symptom tracker, with an average completion rate of 78% of days enrolled.

Based on feedback from the consumer group, we included an option for participants to click a button on the homepage if they had no symptoms to record—effectively a score of 0 across all symptoms. Four participants in phase 2 used this option and recorded 2, 3, 7, and 9 days, respectively, where they had “no symptoms to record.”

#### Recording of Symptoms as Severe: Phase 2

Symptoms were recorded as being severe (8 or above) on individual symptoms a total of 22 times: nausea (n=2), vomiting (n=1), diarrhea (n=2), constipation (n=4), mucositis (n=1), fatigue (n=8), and distress (n=4). In phase 2, 16 red alerts were recorded: constipation (n=2), diarrhea (n=2), distress (n=5), nausea (n=2), vomiting (n=5). The red alerts were generated by 5 of 7 (71%) phase 2 participants. Four people generated multiple red alerts: 1 participant generated 5 separate red alerts for vomiting (n=3), constipation (n=1), and distress (n=1); 1 participant generated 4 separate red alerts for vomiting (n=2), constipation (n=1), distress (n=1), and nausea (n=1); 2 participants generated 2 red alerts for distress (n=3) and diarrhea (n=1); and one participant generated 1 red alert for vomiting (n=1). In addition, 7 (44%) of the 16 reported red alerts also contained at least 1 or 2 amber alerts for other symptoms, including constipation (n=2), distress (n=4), fatigue (n=3), and mucositis (n=1).

#### Generation of Red Alerts: Phase 2

The number of days between treatment and symptom severity triggering a red alert varied between 0 (ie, the same day as chemotherapy) and 11 days. Two of the red alerts also included a report of the participant being admitted to hospital. In addition, 2 hospital admissions were reported following the generation of an amber alert.

#### Visits to the Website and Completion of the Symptom Tracker: Phase 3

In phase 3, the number of times the symptom tracker was completed ranged from 21 to 106 times and days enrolled in SAM ranged from 45 to 117 days. The number of missed days ranged from 0 to 87 days, and completion rate ranged from 21% to 100%, with an average of 59%. Nine participants used the “no symptoms to report” button in phase 3 on between 1 and 10 days: 10 days (n=1), 5 days (n=2), 4 days (n=2), 3 days (n=1), 2 days (n=1), and 1 day (n=2).

#### Recording of Symptoms as Severe: Phase 3

Symptoms were recorded as being severe (8 or above) on individual symptoms a total of 78 times: nausea (n=6), vomiting (n=5), diarrhea (n=8), constipation (n=3), mucositis (n=8), neuropathy (n=16), and fatigue (n=32). Three participants did not record any symptoms of 8 or above and 4 participants only recorded 1 symptom at one time point as 8 or above.

#### Generation of Red Alerts: Phase 3

The number of valid red alerts generated during phase 3 was 38: constipation (n=2), diarrhea (n=13), distress (n=5), nausea (n=7), neuropathy (n=2), high temperature (n=2), and vomiting (n=7). As per the study protocol, these red alerts were forwarded to the medical oncology CNM within 1 to 3 days of being received by the research team in case additional follow-up was required. Two additional red alerts were excluded after being identified as incorrect entries (ie, temperature recorded as 367 instead of 36.7 and 63.8 instead of 36.8).

The 38 red alerts were generated by 12 participants. Seven participants generated multiple red alerts: 6 alerts (n=2), 5 alerts (n=2), 4 alerts (n=2), and 3 alerts (n=1). The remaining 5 participants each generated 1 alert. In addition, 15 (39%) of the 38 reported red alerts also contained between 1 and 3 amber alerts for other symptoms: constipation (n=2), distress (n=6), fatigue (n=5), mucositis (n=4), neuropathy (n=3), and vomiting (n=3).

The number of days between treatment and symptom severity triggering a red alert ranged between 0 (ie, the same day as chemotherapy) and 26 days. Three participants also reported a hospital visit and/or stay at the same time as their red alert. Two of these participants also advised that their chemotherapy cycles were delayed by 1 to 2 weeks due to neutropenia. Four hospital admissions were reported following the recording of amber alerts.

#### Use of Resource Pages

The resource pages were not accessed regularly by participants. In phase 2, 4 participants accessed resource pages and in phase 3, 6 participants accessed resource pages. The participants who accessed resource pages visited a variety of pages rather than one or two pages. No link between symptom experience or severity of symptoms and accessing resource pages was found.

### Questionnaire Data for Phase 3 Participants

#### Mental Health: Anxiety and Depression

The frequency of self-reported levels of anxiety and depression (Table 2) show that at each time period, the majority of phase 3 study participants were experiencing a clinical level of anxiety and a borderline clinical level of depression, which increased (from 53% to 70.5%) and decreased (from 80% to 47%), respectively, over time. The statistical significance of these changes in anxiety and depression could not be reliably determined due to low frequencies across each of the 4 time points.

**Table 2:** Frequencies for mental health categories and median scores for physical and psychological symptom burden and self-efficacy at each time point for phase 3 participants (n=17).

Variables				Time point			
				Baseline	Pre cycle 2	Pre cycle 3	Pre cycle 4
**Mental health, n (%)**							
	**Anxiety**						
		Normal range		3 (18)	2 (12)	1 (6)	2 (12)
		Borderline clinical		5 (29)	7 (41)	3 (18)	3 (17)
		Clinical		9 (53)	8 (47)	13 (76)	12 (71)
		Total		17 (100)	17 (100)	17 (100)	17 (100)
	**Depression**						
		Normal range		2 (13)	3 (18)	3 (19)	6 (35)
		Borderline clinical		12 (80)	14 (82)	9 (56)	8 (47)
		Clinical		1 (7)	0 (0)	4 (25)	3 (18)
		Total		15 (100)	17 (100)	16 (100)	17 (100)
**Symptom burden, median score**							
	Physical symptom distress			36.0	40.0	44.0	48.0
	Psychological symptom distress			13.0	11.5	12.0	13.0
	Overall valuation of life			2.0	2.5	2.5	2.0
Self-efficacy, median score				92.1	105.1	117.1	113.6

#### Symptom Burden

Median scores at each time point for physical symptom distress, psychological symptom distress, and overall valuation of life indicate that participants reported a relatively low level of symptom distress and a good overall valuation of life across the time points (Table 2). The level of physical symptom distress did significantly increase over time (Friedman χ^2^_3_=19.1, *P*<.001; n=14) while the level of psychological symptom distress (Friedman χ^2^_3_=7.4, *P*=.06; n=16) and valuation of life (Friedman χ^2^_3_=2.7, *P*=.44; n=16) did not vary significantly over time.

#### Self-Efficacy

Reports of self-efficacy were relatively high among the phase 3 participants at each time point (Table 2). However, the increase in median self-efficacy scores from baseline over the treatment cycle was not statistically significant (Friedman χ^2^_3_=0.4, *P*=.94; n=12).

#### Quality of Life

Data on the participants’ quality of life, as measured by the FACT-B (version 4) and FACT-C (version 4), were not reported, as full scale scores for approximately 60% of participants at each time point could not be calculated because of missing values. Missing values were spread across a number of scale items, although the item with the highest number of missing responses concerned the participant’s satisfaction with their sex life. Up to 58% of respondents opted not to respond to this item. This may be because participants considered the issue too personal to disclose or because they considered a response to the item to be unrelated to their illness. Future studies of the self-reported quality of life of persons undergoing cancer treatment might consider using an alternative, shorter validated and reliable measure and ensure that participants cannot opt out of responding to items (eg, use a forced-choice survey format).

### Interview Data

Fifteen interviews were completed: 4 in phase 1 and 11 in phase 2. The findings from phase 1 were comparable with those identified in phase 2 and the combined data. The key findings were ability to use technology and benefits of using the system and recommendations.

#### Ability to Use Technology

Participants received training in accessing and using the site. All described the training as adequate and the site as straightforward and easy to use:

Oh definitely. Just basic. That was good. Yes. And just so it was quick to go through it as well. And for me with no computer skills that’s saying something.

P204

Participants accessed the site on a range of devices—computer, smartphone, or tablet—and nearly all participants used the same device throughout the study. Participants reported being able to connect to the site easily, although many reported having trouble activating the option to remain logged into the webpage. Once this was explained, no further issues were reported.

No, nothing major. I forgot the passwords a couple of times and got myself into a bit of a muck. But that was my problem. But no I managed to get there.

P218

Participants reported that the site was well designed, straightforward, and easy to use. All participants described the scale (0-10) as easy to navigate, and all participants stated that they felt a scale was the best way to measure symptom severity, rather than a method such as emojis.

I use them (emojis). I use them in my texts, and I use them, but I’m not sure they are completely medically appropriate.

P208

#### Key Benefits of Using the System

All participants reported the key benefits of the system as being made aware of their symptoms and changes in symptoms over time, as well as being able to account for symptoms.

Basically it kept you where you were. What’s going on, knowing all your symptoms, keeping up with things and keeping up with you know the side effects. Yeah that was important to me. I've noticed now because I haven’t been using it. It’s hard to keep track of where I’m at what’s happening.

P204

Participants were required to enter data on the SAM site once a day. Although data collected directly from the website suggests not all participants completed daily entries for the duration of the study period, everyone described a daily level of engagement as repetitive but acceptable.

It’s repetitive of course but it is what it is. But I found it not too hard to fill in. No not at all.

P206

Remembering to log in was sometimes an issue, with participants suggesting they either forgot or did not always feel well enough. Even so, using the system did not appear to have an impact on daily routines or cause distress.

Sometimes it was because I was feeling really rough and other times it was that I basically forgot. I should have done it…and I suppose if I hadn’t done it one day I thought I might have thought ‘did I do it’? I couldn’t remember whether I’d done it as well sometimes. I suppose that sometimes I’d go in and I’d do it—put my symptoms in and then I don’t think it logged it because it kinda logged me out and I had to log back in and then I’d repeat what I’ve just done.

P221

Participants mostly described remaining engaged with the self-care messages that were returned and continued to see them as positive and to read them.

I can’t remember the ones that popped up off the top of my head but they were useful. It was like, it was good to know. Oh yeah. Great I haven't been nauseous today. That's nice. Like that was good, like positive reinforcement that things are getting a bit easier. So that was good.

P211

Overall, participants were positive about the alert facility of the system, reporting that they felt “secure” in the knowledge that their data were being tracked and they had a record. Participants were aware that their symptoms were not being monitored by clinicians; however, they were aware that alerts would be forwarded to the CNM, who in turn would make a note of the alert on the patient’s file and follow them up as required. This resulted in a follow-up call made to the participant or their family, which was received positively.

My husband got a call from the medical oncology nurse after I had been admitted to hospital. This gave him relief that I was being followed up.

Pilot, Respondent A

As I say some people at the other end reacted when it flagged up a possible problem so I was quite impressed.

P206

All participants interviewed described feeling reassured that their symptoms were being tracked.

I found that the whole thing was very useful keeping a track on myself as well as knowing where I’m at.

P204

The information was you know that it sort of reassured me that things were going probably as they should do.

P205

Participants described having information collated in one place and the ability to review patterns helpful.

Yes they were useful, they were a little bit um, what’s the word. They would sort of ease your mind a wee bit when you went through them to say well things aren’t quite as bad as you might think they are.

P205

Yeah I’ll go back and have a look and then I see the little patterns. When you are worse and such, when you come good you can follow it that way.

P207

Several participants described following recommendations generated in the daily symptom tracker to contact a clinician or a medical oncology team member, or to visit the emergency department based on the feedback.

Even with the green, I was more like oh okay I’ll take that in note and I’ll suggest it to my specialist.

P204

I think the three different colors (symptom tracker web page) was definitely good. Obviously orange was sort of like I’ve really got to pay attention. I did ring the nurses a few times if I was getting a randomly different side effect.

P211

Yes. I had to go in—high temp...I think it did tell me like bang—This is high. Like consult. Don’t wait for the next day or see how you are in the morning.

P207

Once I hit the orange then it was like yeah I’ll give them a call. So it was good to have that as a reference.

P211

A number of participants described the system as supporting them to manage their symptoms more effectively:

Yes there would have been times where I went Ah don’t worry I’ll feel okay I just won’t bother with my temperature. Yeah. And I think that was a big eye opener…So it made me go stop, rest and recheck myself.

P204

I think yeah the color coding thing, definitely. So if I was in the orange or the red it definitely causes you to do something. It definitely causes you to action something. So yes definitely in that sense.

P211

The ability to assess symptoms over time was valued:

Just the fact that you can go back and just having a little bit of a good look at the history you know that sometimes you can’t quite remember sometimes. When you had a bad day or something you can go back and go you can see your peaks and troughs—these things that you’ve selected. I’ve gone back and had a look and see how it goes up and down. [referring to graphs] cos you forget sometimes when your good days are and your bad days.

P207

#### Recommendations

While many of the participants described the symptoms graphs as helpful, it was clear that the interpretation of the graphs became more difficult as data entry increased. One respondent also reported that the date of entry did not appear on the printout, while other participants found the graphs difficult to view on a smartphone. This is an area for attention in future versions of the program.

The pilot study found that the resources page was accessed at the beginning of the chemotherapy cycle but not much thereafter. In phase 3, to encourage continued engagement with the resources page, additional reminders were added to the daily summary page:

Well there were links and information that you could actually access direct from there. Sometimes I was just too tired to follow it through. But when I was able I appreciated the fact that the information was there. So I’ve actually kept the stuff in mind to take action. I’ve actually printed some of this stuff out so I know what to do if I get a um, what to watch out for.

P218

Several participants referred to forgetting to enter data or feeling too ill to enter data. We added a button on the front page so that participants could indicate that they hadn’t forgotten but did not feel like completing the scales. Some participants suggested the ability to enter data retrospectively would be valuable and this could be a feature in a future version of the program.

The thing that I actually found the most frustrating to be honest, was the fact that and especially initially um you couldn’t go back. Like the first day I logged in and I couldn’t actually go back to like the couple of days before that and put in, like I’d noted down what my symptom were but I couldn’t go back because of it. Like you couldn’t do it for a specific date. I think would’ve been helpful for you guys and for me.

P208

During the pilot phase, we identified incorrect data entries when participants forgot to move the slider, which had a default setting of “5”, resulting in an amber alert. As a result, all sliders were set to 0 as a starting point. Some participants suggested that it would have been good if they had the ability to go back and edit entered data, as sometimes the slider would land on a number they didn’t intend to submit:

But again—also being able to edit it because sometimes I accidentally pressed like it went to 10 because I scrolled up and I accidentally pressed it not realizing yet because it would look like I’ve hit a 10 but I can’t go back and edit it.

P211

Some participants asked for more information on specific issues, including the use of pain killers, a specific focus on the first week of treatment when symptoms were at their worst, and the use of supplements:

Yeah. And painkillers as well because at the moment I’m just going from one lot of pain killers to another lot of pain killers and they don’t really tell you much on the side effects…Or other supplements that can be added like Sustagen and things like that. You know I’ve had to remind myself I can take that.

P204

Some participants suggested providing different daily messages:

Yeah definitely. I can’t remember what they say exactly but from memory it was just the first time I read it either. Yeah. And then I noticed after a few days was the same thing so I just I was okay with that. And when it changed color I read it again. Come on. OK it’s like something different again. I think that, like I said before the more personable thing that’s definitely something to look at.

P211

Participants suggested that setting up the program as an app would be helpful in relation to accessing the program directly from their smartphone and the ability of the app to generate push notifications as reminders to complete the symptom tracker. Overall, participants reported positive experiences and many believed the system has great potential to be further developed.

## Discussion

### Principal Results

Our results demonstrated that participants involved in this study were positive about their experience of using SAM to monitor and manage their chemotherapy-related symptoms. Participants found SAM to be helpful in supporting them to manage symptoms and described feeling confident in accessing and using the site. Participants reported that using SAM increased feelings of reassurance and security related to awareness of symptoms and changes on a daily basis. The generation of an alert when symptoms were moderate or high was overwhelmingly viewed as positive by participants, who described acting on the advice given, and participants were impressed when the oncology team followed up with them. The interactive aspects of SAM highlight the ability of technology to be used for purposes beyond data collection. The system has established procedures to both generate feedback and promote early intervention. Based on their experiences, participants could see potential for the development of SAM, in terms of both the functionality of the system and developments within the health care system. Suggestions for developments include the addition of an alarm feature to remind participants to complete the symptom scales, the ability to enter missed data and edit data entered in error, and an option to allow the report of any additional symptoms experienced that were not covered by the core symptoms reported. The functionality of the system, positive feedback from patients, and refinements of the system based on feedback from participants, consumers, and the advisory group support recommendations for the further development of the system and its use within health care services.

### Limitations

The limitations of the study include that it was set up as a pilot study and as such the sample size was small and no comparison group data were collected. Nearly one-half of the individuals who were approached to participate declined. The most common reason given was feeling overwhelmed with their diagnosis and upcoming treatment. Data were collected over a limited time period, and the role of a system like SAM over a longer time period—even posttreatment—in the improvement in supportive care needs remains an area for exploration. Participation was restricted to 2 tumor groups and the applicability of the system to people with similar symptom profiles is likely but cannot be confirmed. Future research with a larger sample of patients receiving adjuvant chemotherapy following breast or colorectal cancer and a comparison group is recommended. Exploratory studies to adapt the SAM system for people with advanced disease and those receiving other treatment modalities (eg, immunotherapy) are recommended.

### Conclusions

This study indicated that people receiving postoperative chemotherapy for breast or colorectal cancer had positive perceptions of and experiences using SAM to monitor and manage chemotherapy-related toxicity. The remote monitoring of symptoms and an alerting system helped to ensure that people who were experiencing symptoms were identified early and that participants were facilitated to seek timely intervention. This has the potential to reduce both the severity and duration of the symptoms experienced, promoting a system of care that is anticipatory and preventative rather than reactive. This serves to enhance patient safety as a direct line of communication between the patient, cancer specialists, and the general practitioner, and provides patients with access to evidence-based, real-time feedback based on their experience of symptom severity as required.

## Appendix

Multimedia Appendix 1 Log-in screen on a smartphone.

Multimedia Appendix 2 Log-in screen on a tablet.

Multimedia Appendix 3 Self-care messages.

Multimedia Appendix 4 Symptom history.

Multimedia Appendix 5 Symptom tracker.

## Abbreviations

CNM: clinical nurse manager
FACT-B: Functional Assessment of Cancer Therapy-Breast
FACT-C: Functional Assessment of Cancer Therapy-Colorectal
HADS: Hospital Anxiety and Depression Scale
RSCL: Rotterdam Symptom Checklist
SAM: Symptom Assessment and Management
SUPPH: Strategies Used by Patients to Promote Health
