# Supplementary figures and images for: Evaluation of a Remote Symptom Assessment and Management (SAM) System for People Receiving Adjuvant Chemotherapy for Breast or Colorectal Cancer: Mixed Methods Study
Source: JMIR Cancer. 2020 Dec 7;6(2):e22825. doi: 10.2196/22825 (PMC7752534; doi:10.2196/22825)

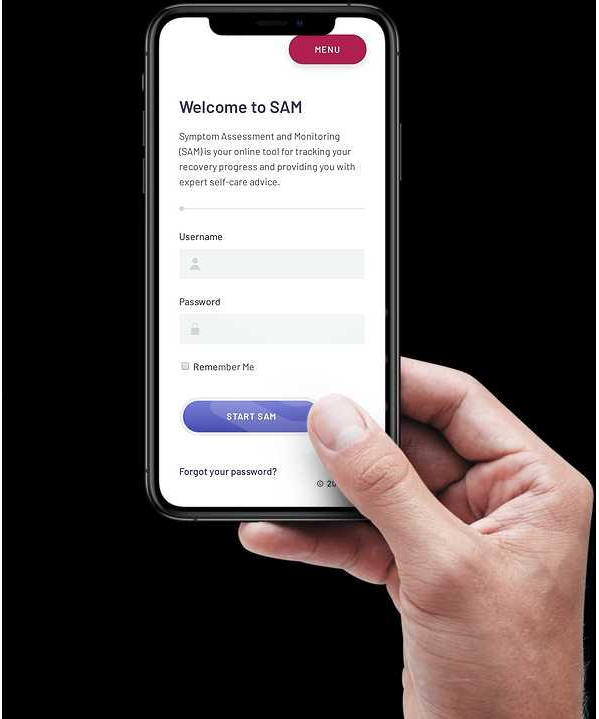

Supplement: Multimedia Appendix 1 [file cancer_v6i2e22825_app1.png]

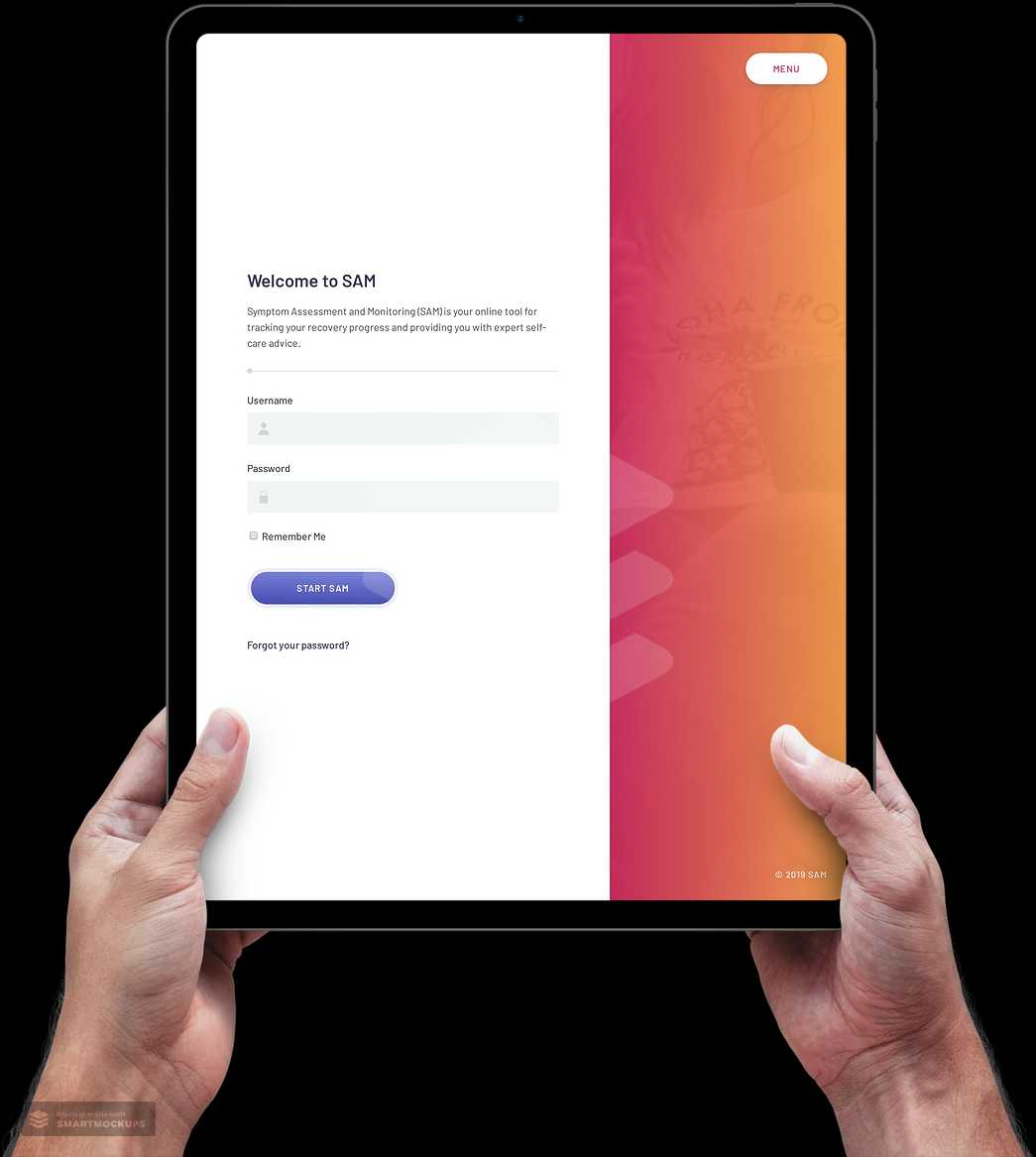

Supplement: Multimedia Appendix 2 [file cancer_v6i2e22825_app2.png]

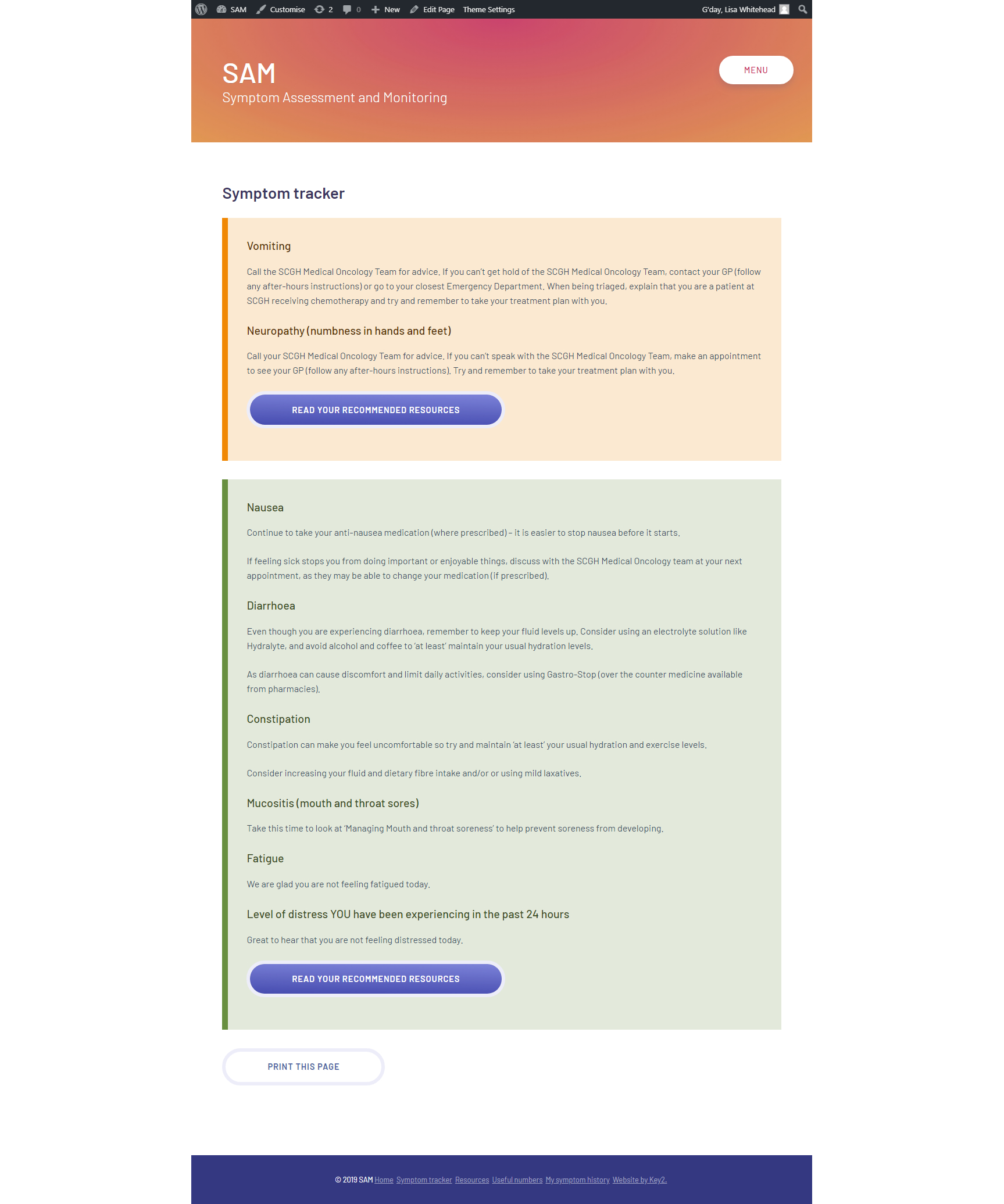

Supplement: Multimedia Appendix 3 [file cancer_v6i2e22825_app3.png]

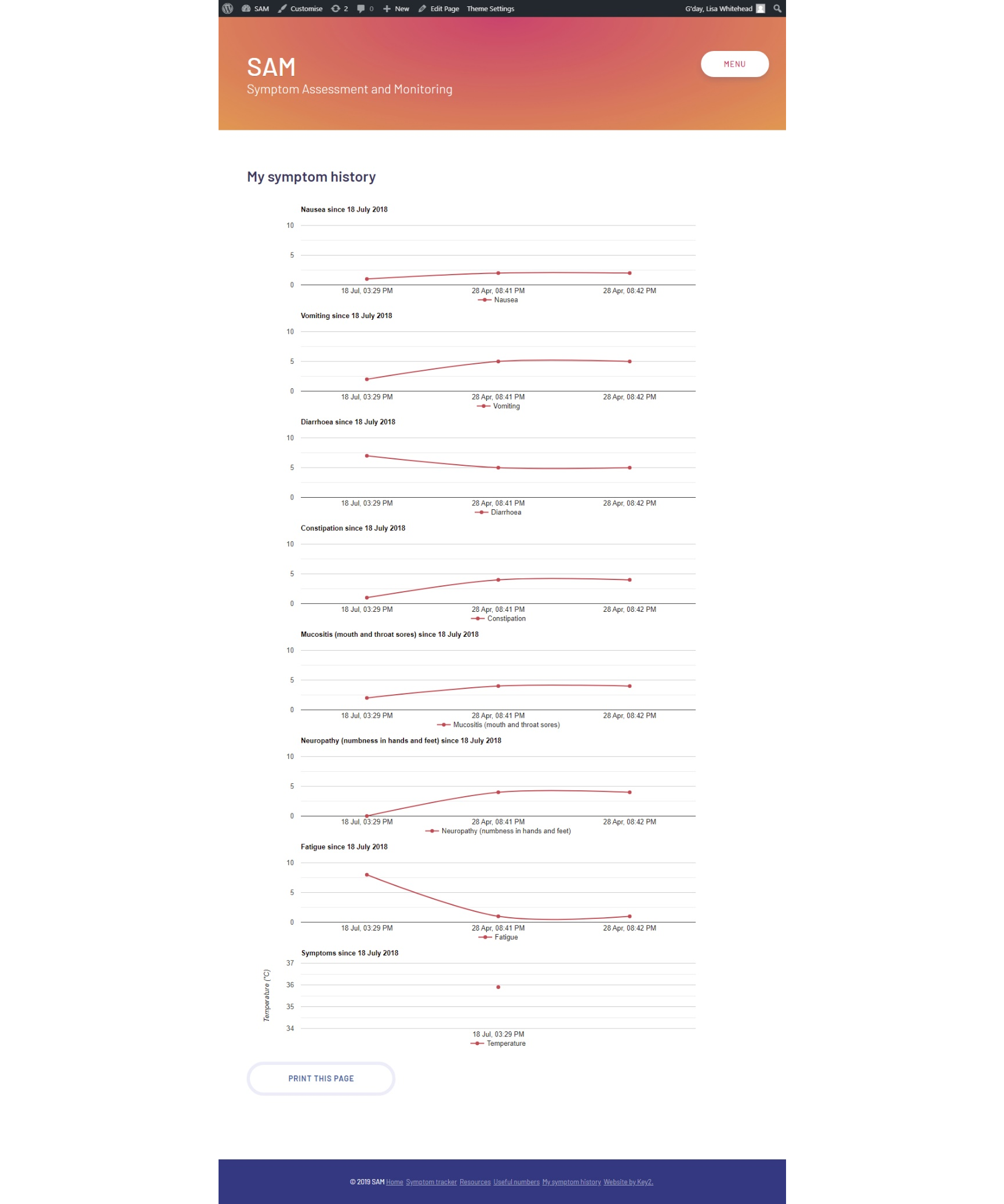

Supplement: Multimedia Appendix 4 [file cancer_v6i2e22825_app4.png]

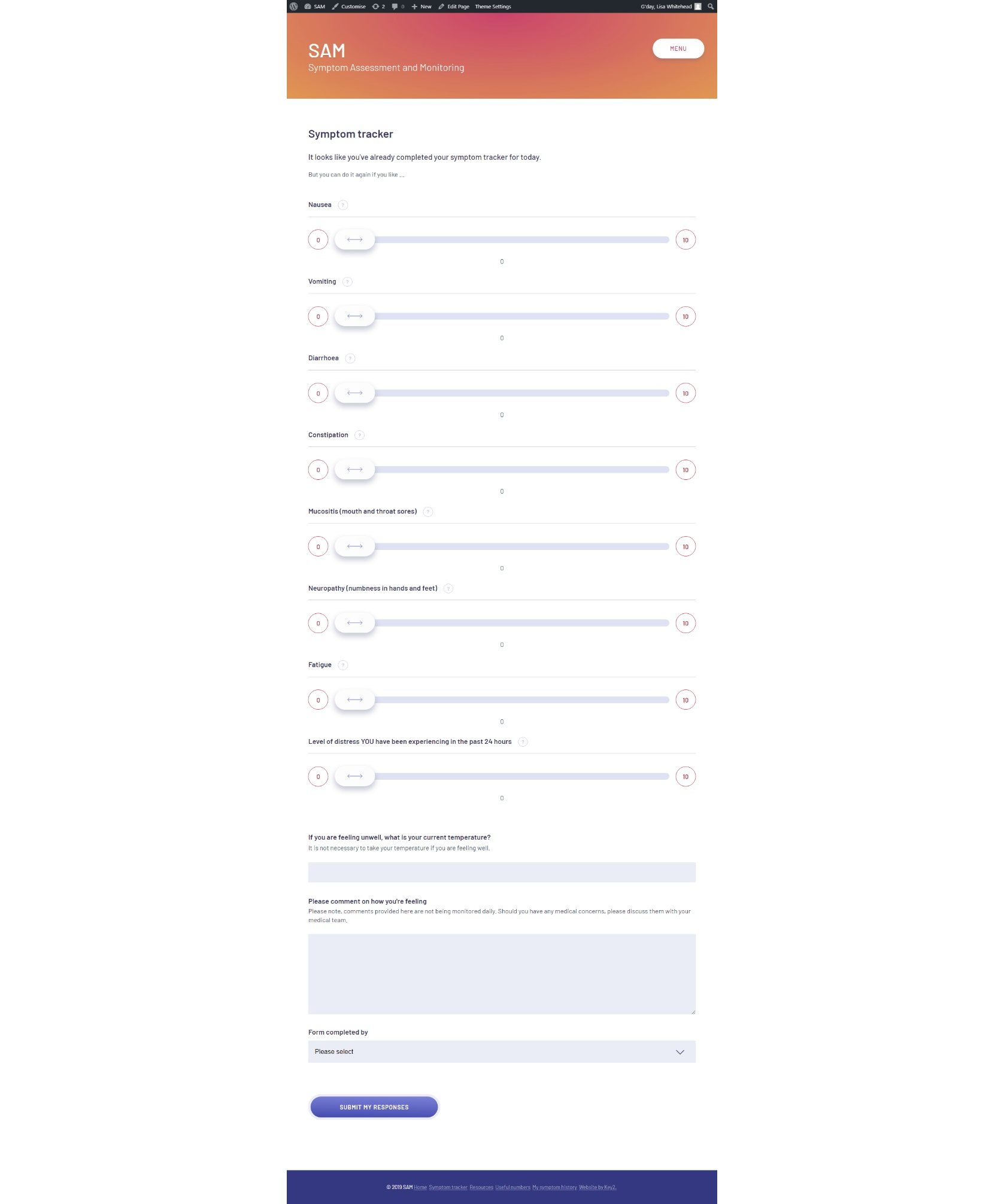

Supplement: Multimedia Appendix 5 [file cancer_v6i2e22825_app5.png]
